# Risk of CKD among patients with DM taking diuretics or SGLT2i: a retrospective cohort study in Taiwan

**DOI:** 10.1186/s40360-024-00745-7

**Published:** 2024-03-05

**Authors:** Han-Jie Lin, Pin-Yang Shih, Stella Chin-Shaw Tsai, Wu-Lung Chuang, Tsai-Ling Hsieh, Heng-Jun Lin, Teng-Shun Yu, Fuu-Jen Tsai, Chiu-Ying Chen, Kuang-Hsi Chang

**Affiliations:** 1 Department of Otolaryngology, Tungs’ Taichung MetroHarbor Hospital, 435 Taichung, Taiwan; 2 Department of Public Health, China Medical University, 406040 Taichung City, Taiwan; 3 Rong Hsing Research Center for Translational Medicine, College of Life Sciences, National Chung Hsing University, 402 Taichung, Taiwan; 4 Division of Endocrinology and Metabolism, Department of Internal Medicine, Changhua Christian Hospital, 500 Changhua City, Taiwan; 5 Division of Endocrinology and Metabolism, Department of Internal Medicine, Lukang Christian Hospital, 505 Changhua County, Taiwan; 6 Department of Medical Research, Tungs’ Taichung MetroHarbor Hospital, 435 Taichung, Taiwan; 7 Management Office for Health Data, China Medical University Hospital, Taichung, Taiwan; 8 College of Medicine, China Medical University, Taichung, Taiwan; 9 School of Chinese Medicine, College of Chinese Medicine, China Medical University, 404 Taichung, Taiwan; 10 Department of Medical Research, China Medical University Hospital, China Medical University, 404 Taichung, Taiwan; 11 Division of Medical Genetics, China Medical University Children’s Hospital, 404 Taichung, Taiwan; 12 Department of Biotechnology and Bioinformatics, Asia University, 413 Taichung, Taiwan; 13 Center for General Education, China Medical University, 404 Taichung, Taiwan; 14 General Education Center, Jen-Teh Junior College of Medicine, Nursing and Management, 356 Miaoli, Taiwan

**Keywords:** Diuretics, Sodium-glucose cotransporter-2 inhibitors (SGLT2i), Chronic kidney disease (CKD), National Health Insurance Research Database (NHIRD).

## Abstract

**Background:**

This study aimed to evaluate the long-term risk of CKD and renal function declines using a combination of diuretics and SGLT2i.

**Methods:**

We selected the data of subjects who had at least two outpatient records or at least one inpatient record for DM treatment as the DM group from the National Health Insurance Research Database (NHIRD). Patients receiving versus not receiving SGLT2i were defined as the SGLT2i and non-SGLT2i cohorts, respectively. The patients in the two groups were matched 1:1 through propensity score matching based on age, sex, year of index date, and comorbidities.

**Results:**

The diuretics-only group had a higher risk of CKD (aHR, 2.46; 95% CI, 1.68–3.61) compared to the neither SGLT2i nor diuretics group, while the both SGLT2i and diuretics group and the SGLT2i only group had lower risks (aHR, 0.45, 95% CI, 0.32–0.63; aHR, 0.26, 95% CI, 0.17–0.40) than the diuretics-only group. The SGLT2i-only group had a lower risk (aHR, 0.58, 95% CI, 0.36–0.94) than the both SGLT2i and diuretics group.

**Conclusion:**

This study indicates that diuretics could raise the risk of CKD in diabetic patients, but when used in combination with SGLT2i, they continue to offer protection against CKD.

## Introduction

Millions of people worldwide have chronic kidney disease (CKD) [1], a common complication in diabetes mellitus (DM) and hypertension (HT) [2, 3]. The incidence of CKD is higher in people with older age, HT, and DM [4, 5]. Low kidney function and high albumin levels are associated with the incidence of heart failure [6–8].

Diuretics are often used to treat heart failure, pulmonary edema, and general edema. One study showed that the use of diuretics is associated with acute kidney injury (AKI), especially in patients with older age and taking higher dosages. The combination of diuretics and other drugs, e.g., nonsteroidal anti-inflammatory drugs, angiotensin-converting enzyme inhibitors, antibiotics, and contrast media, may also increase an individual’s risk of AKI [9].

Another study revealed that diuretics use is associated with the incidence of end-stage renal disease (ESRD) and a greater decrease rate in estimated glomerular filtration rate (eGFR) compared with other anti-hypertensive drug use [10]. Sodium-glucose cotransporter-2 inhibitors (SGLT2i) can also increase fluids and urine glucose excretion output. SGLT2i may decrease the progression and the rate of renal function decline in CKD patients with or without diabetes [11]. Few studies to date have clarified whether the combined use of diuretics and SGLT2i may be beneficial or risky for patients with CKD. This study aimed to evaluate the long-term risk of CKD and renal function declines with the use of a combination of diuretics and SGLT2i.

## Method

### Data source

The data for this retrospective cohort study were sourced from the Longitudinal Generation Tracking Database (LGTD), a derivative of the extensive National Health Insurance Research Database (NHIRD). The NHIRD was established in Taiwan in 1995 as a single-payer national health insurance program. The LGTD was created by randomly selecting approximately 2 million insured individuals based on age and sex distributions from the NHIRD. These selected claims data were rigorously encrypted and anonymized to safeguard patient privacy.

The claims data within the LGTD encompassed a wide range of information, including demographic details, inpatient and outpatient visit dates, diagnostic codes for diseases in accordance with the International Classification of Diseases, 9th and 10th editions, clinical modification (ICD-9-CM and ICD-10-CM), and anatomical therapeutic chemical codes for medications.

The Institutional Review Board of China Medical University (CMUH110-REC3-133(CR-1)) approved this study. Importantly, informed consent was not required from the insured individuals since the data within the LGTD had already been meticulously encrypted and anonymized to protect their privacy.

### Ethics statement

The NHIRD secures patient personal data via encryption to guarantee privacy protection and provides researchers with anonymous identification numbers connected to essential claims data, including gender, birthdate, medical services, and prescriptions. As a result, accessing the NHIRD does not require patient consent. This research project obtained exemption approval from the Institutional Review Board (IRB) at China Medical University (Approval Number CMUH109-REC2-031 (CR2)), with the IRB specifically waiving the consent requirement. All the procedures were followed in accordance with the relevant guidelines and regulations.

### Study population

We selected the data of subjects who had at least two outpatient records or at least one inpatient record for DM treatment as the DM group. Various comorbidities, considered influential factors in this study, included HT, hyperlipidemia (HL), stroke, asthma, chronic obstructive pulmonary disease (COPD), and disorders related to alcohol consumption. Patients were classified as having these conditions if they received diagnoses for them at least twice in an outpatient clinic or at least once during inpatient care.

**Fig. 1:**
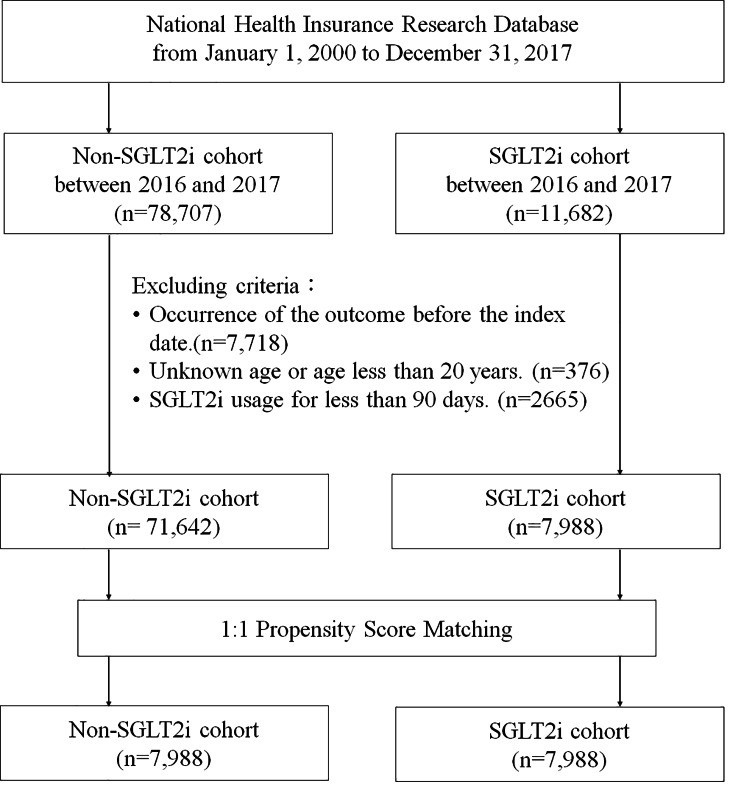
Flowchart of establishing the study cohorts

Patients receiving versus not receiving SGLT2i were defined as the SGLT2i and non-SGLT2i groups, respectively. In the SGLT2i group, the index date was defined as the first date that SGLT2i was administered. In the non-SGLT2i group, the index date was defined as a random date during the study. Patients for whom outcomes occurred before the index date, those with missing age data, < 20 years of age, and those with SGLT2i usage for < 90 days were excluded. The SGLT2i and non-SGLT2i groups were paired in a 1:1 ratio using propensity score matching, considering variables such as sex, age, year of index date, diuretic usage, and presence of comorbidities including HT, HL, stroke, asthma, COPD, and alcohol-related disorder (ARD). Flowchart shows recruitment of subjects from NHIRD (Fig. 1). Figure 1 shows the flowchart of establishing the study cohorts.

### Outcome and comorbidities

The primary study outcome was CKD/ESRD (ICD-9-CM code 585; ICD-10-CM code N18). We considered the comorbidities mentioned below as relevant if they occurred before the index date: HT (ICD-9-CM codes 401–405; ICD-10-CM codes I10, I11, I12, I13, I15, N26.2), HL (ICD-9-CM code 272; ICD-10-CM codes E71.30, E75.21, E75.22, E75.24, E75.3, E75.5, E75.6, E77, E78.0, E78.1, E78.2, E78.3, E78.4, E78.5, E78.6, E78.70, E78.79, E78.8, and E78.9), asthma (ICD-9-CM code 493; ICD-10-CM code J45), COPD (ICD-9-CM codes 490–496 and 504–506; ICD-10-CM codes J40–J47 and J64–J68), and alcohol-related disorder (ICD-9-CM codes 291, 303, 305.0, 571.0–571.3, 790.3, V11.3, and V79.1; ICD-10-CM codes F10, K70, R78.0, and Z65.8).

### Statistical methods

Continuous variables, such as age and follow-up period, are expressed as mean and standard error, and differences were tested using Student’s t-test. Categorical variables, such as sex, age group, and comorbidities, are expressed as percentages, and differences were tested using the chi-squared test. Incidence rate (IR) and person-years are commonly used epidemiological measures to describe the occurrence of CKD and ESRD over time. Cox regression analysis utilized follow-up time, exposure, and outcome to calculate the hazard ratio between the two groups. All statistical analyses were performed using SAS version 9.4 (SAS Institute, Inc., Cary, NC, USA), and differences with *p* < 0.05 were considered to be statistically significant.

## Results

As shown in Table 1, the study included 15,976 patients aged ≥ 20 years. Of them, 7,988 (50%) received SGLT2i; 4,445 were male (55.7%) and 3,543 were female (44.3%). The mean (standard deviation) age of the SGLT2i and non-SGLT2i cohort was 57.66 (11.79) and 57.81 (11.75) years, respectively. The top two comorbidities in this study were HT and HL. The mean (standard deviation) follow-up period differed significantly between the two cohorts: 0.94 (0.41) years in the non-SGLT2i cohort versus 0.91 (0.58) years in the SGLT2i cohort.

**Table 1:** Demographic characteristics, comorbidities, and medications of patients with DM taking or not taking SGLT2i

	Non-SGLT2i group (*n* = 7,988)	SGLT2i group (*n* = 7,988)	*P*
	*n* (%) / Mean ± SD	*n* (%) / Mean ± SD	
Male sex	4,458 (55.81)	4,445 (55.65)	0.836
Age, years			0.861
20–39	532 (6.66)	549 (6.87)	
40–59	3,780 (47.32)	3,778 (47.30)	
≥ 60	3,676 (46.02)	3,661 (45.83)	
Mean	57.81 ± 11.75	57.66 ± 11.79	0.418
Comorbidities			
HT	6,184 (77.42)	6,167 (77.20)	0.748
HL	6,972 (87.28)	6,979 (87.37)	0.868
Asthma	1,371 (17.16)	1,384 (17.33)	0.785
COPD	1,557 (19.49)	1,559 (19.52)	0.968
ARD	289 (3.62)	296 (3.71)	0.768
Follow-up period, years	0.91 ± 0.58	0.94 ± 0.41	< 0.001

ARD, alcohol-related disorder; COPD, chronic obstructive pulmonary disease; HL, hyperlipidemia; HT, hypertension

Patients were stratified according to demographics and comorbidities (Table 2). Across all ages, patients who received SGLT2i had a lower IR for CKD/ESRD than those who did not. An examination of the adjusted HR (aHR) yielded the following results: in male patients, those ≥ 60 years of age, and patients with comorbidities, such as HT, HL, asthma, and COPD, the SGLT2i group demonstrated a significantly lower risk for CKD/ESRD than the non-SGLT2i group (all aHR < 1, *p* < 0.05).

**Table 2:** IR and HR of CKD/ESRD associated with SGLT2i use in patients with DM stratified by demographics and comorbidities

		IR		cHR (95% CI)	aHR (95% CI)
		Non-SGLT2i	SGLT2i		
Sex	Female	21.69	16.41	0.64 (0.42, 0.98)*	0.71 (0.43, 1.17)
	Male	39.09	11.9	0.42 (0.29, 0.62)***	0.29 (0.17, 0.48)***
Age, years	20–39	10.26	9.45	1.28 (0.21, 7.67)	0.90 (0.06, 14.58)
	40–59	20.87	12.01	0.52 (0.32, 0.84)**	0.54 (0.29, 1.00)
	≥ 60	37.76	15.9	0.48 (0.34, 0.69)***	0.40 (0.26, 0.62)***
Comorbidities					
HT	No	20.99	12.74	0.73 (0.33, 1.61)	0.58 (0.16, 2.07)
	Yes	31.15	14.32	0.48 (0.36, 0.65)***	0.44 (0.31, 0.63)***
HL	No	50.61	2.77	0.27 (0.12, 0.62)**	0.05 (0.01, 0.36)**
	Yes	27.93	15.53	0.56 (0.41, 0.75)***	0.53 (0.37, 0.76)***
Stroke	No	27.88	14.51	0.55 (0.40, 0.76)***	0.50 (0.34, 0.75)***
	Yes	38.61	13.01	0.37 (0.19, 0.69)**	0.31 (0.15, 0.64)**
Asthma	No	30.22	14.64	0.54 (0.40, 0.74)***	0.46 (0.31, 0.67)***
	Yes	30.39	12.44	0.36 (0.18, 0.73)**	0.40 (0.18, 0.89)*
COPD	No	24.2	12.71	0.59 (0.42, 0.82)**	0.51 (0.33, 0.79)**
	Yes	49.2	18.4	0.36 (0.21, 0.61)***	0.35 (0.20, 0.64)***
ARD	No	29.3	13.26	0.49 (0.37, 0.67)***	0.43 (0.30, 0.62)***
	Yes	52.89	34.49	0.63 (0.25, 1.56)	0.55 (0.17, 1.76)

aHR, adjusted hazard ratio; ARD, alcohol-related disorder; cHR, crude hazard ratio; COPD, chronic obstructive pulmonary disease; HL, hyperlipidemia; HR, hazard ratio; HT, hypertension; IR, incidence rate (per 1000 person-years); SGLT2i, sodium-glucose cotransporter-2 inhibitors; *p < 0.05, **p < 0.01, ***p < 0.001

As shown in Table 3, patients who received SGLT2i over 196 days had significantly lower risk of CKD/ESRD than patients who did not receive SGLT2i. Compared to patients without receiving SGLT2i, the aHR equaled 0.52 in patients receiving SGLT2i within 197–448 days. Then, the aHR equaled 0.05 in patients receiving SGLT2i over 448 days.

**Table 3:** IR and HR of CKD/ESRD associated with duration of SGLT2i use among patients with DM

Days	Event	PY	IR	aHR (95% CI)	
None	139	7,292	19.06	1 (Reference)	
90–196	29	1,346	21.54	1.17 (0.78, 1.75)	1 (Reference)
197–448	43	3,632	11.84	0.61 (0.43, 0.86)**	0.52 (0.33, 0.84)**
≥ 448	3	2,563	1.17	0.06 (0.02, 0.18)***	0.05 (0.01, 0.16)***

aHR, adjusted hazard ratio; CKD, chronic kidney disease; days, SGLT2i use duration; ESRD, end-stage renal disease; event, number of patients with CKD; IR, incidence rate (per 1000 person-years); PY, person-years; SGLT2i, sodium-glucose cotransporter-2 inhibitors; *p < 0.05, **p < 0.01, ***p < 0.001

The participants were divided into four groups: neither SGLT2i nor diuretics, diuretics only, both SGLT2i and diuretics, and SGLT2i only. Compared to the neither SGLT2i nor diuretics group, the diuretics only group were at an increased risk of CKD after the control for age, sex, and comorbidities (aHR, 2.46; 95% CI, 1.68–3.61). With the diuretics only group as reference, the aHR were 0.45 (95% CI, 0.32–0.63) and 0.26 (95% CI, 0.17–0.40) in the both SGLT2i and diuretics group and the SGLT2i only group, respectively. Compared to the both SGLT2i and diuretics group, the aHR in the SGLT2i only group was 0.58 (95% CI, 0.36–0.94) after the control for age, sex, and comorbidities (Table 4).

**Table 4:** IR and aHR of CKD/ESRD associated with SGLT2i and diuretics use in patients with DM

SGLT2i or diuretics	Event	PY	IR	aHR (95% CI)		
Neither	40	4,020	9.95	1 (Reference)		
Diuretics only	99	3,272	30.26	2.46 (1.68, 3.61)***	1 (Reference)	
Both	48	3,387	14.17	1.1 (0.71, 1.7)	0.45 (0.32, 0.63)***	1 (Reference)
SGLT2i only	27	4,154	6.5	0.64 (0.39, 1.04)	0.26 (0.17, 0.40)***	0.58 (0.36, 0.94)*

aHR, adjusted hazard ratio; CI, confidence interval; CKD, chronic kidney disease; DM, diabetes mellitus; ESRD, end-stage renal disease; event, number of patients with CKD; IR, incidence rate (per 1000 person-year); PY, person-years; SGLT2i, sodium-glucose cotransporter-2 inhibitors; *p < 0.05, **p < 0.01, ***p < 0.001

## Discussion

The risk of CKD/ESRD was assessed in this study of real-world NHIRD data of patients with T2DM, specifically SGLT2i users versus nonusers. The male-to-female ratio in the two groups was equivalent, and the age distribution in the intermediate (40–59 years) and old (60 + years) age groups was also equal. The population’s mean age was 57 years. HL and HT, which affected roughly 87% and 77% of these patients with DM, respectively, were the most common comorbidities. The average length of follow-up was evenly dispersed for around 10 months (Table 1). The diagnosis of CKD/ESRD was made by screening for ICD-9 code 585, which indicates an eGFR reduction over > 7 days. As a result, using diuretics or SGLT2i raised the aHR of eGFR reduction among patients with DM compared to using neither SGLT2i nor loop diuretics, showing acute kidney damaging effects (Table 2). However, a comparison of the use of SGLT2i and loop diuretics revealed aHR for an eGFR reduction for the diuretics only, both SGLT2i and diuretics, and SGLT2i groups in descending order, demonstrating that SGLT2i provides more favorable renoprotection than loop diuretics.

We also discovered that, among DM patients using diuretics and SGLT2i, the aHR was greater in the first 60 days and decreased as the treatment period progressed (Table 3). As a result, our findings differed from those of previous large-scale clinical trials that correlated long-term SGLT2i use with kidney preservation after the control for age, sex, and other comorbidities such as stroke, HL, and chronic heart failure [12–14]. We did learn, however, that the longer SGLT2i is administered, the lower the risk of an eGFR reduction.

The effects of SGLT2 inhibitors on the kidneys are multifaceted, stemming from extensive glycosuria and natriuresis resulting from their primary sites of action. This leads to hemodynamic and metabolic changes that mediate kidney-protective effects, including the following. First, decreased workload of proximal tubular cells and prevention of abnormal increases in glycolysis, reducing the risk for AKI. Second, lowering of intraglomerular pressure through the activation of tubuloglomerular feedback, along with reductions in blood pressure and tissue sodium content. Third, initiation of nutrient-sensing pathways akin to starvation, activating ketogenesis, increasing autophagy, and restoring carbon flow through the mitochondria without generating reactive oxygen species. Fourth, body weight loss without a reduction in basal metabolic rate, which is attributed to increases in non-shivering thermogenesis. Fifth, favorable alterations in the quantity and characteristics of perirenal fat, leading to decreased release of adipokines that negatively impact the glomerular capillary and signal increased sympathetic outflow [15–17].

This hypoglycemic impact is accompanied by decreased glomerular hyperfiltration, decreased afferent HT, and decreased albuminuria, delaying and even improving diabetic nephropathy. Previous clinical studies of SGLT2i have shown renal benefits via a variety of mechanisms, including reduced glomerular HT, a 30–50% lower incidence of albuminuria, decreased levels of inflammatory mediators (e.g. monocyte chemoattractant protein-1, interleukin-6, nuclear factor-κB, reactive oxygen species), increased proximal natriuresis, 4–6 mmHg lower blood pressure, a lower glycated hemoglobin (HbA1c) level, and a lower body weight [18]. Our data revealed that SGLT2i usage for more than 61 days was associated with a decreased risk of an eGFR reduction, which was supported by prior clinical findings of canagliflozin, empagliflozin, and dapagliflozin, showing the efficacy of eGFR reduction and stabilization in 4 weeks [19–21]. The surge in clinical trials focusing on SGLT2i has rapidly broadened the accepted clinical applications of these agents beyond those in individuals with DM [22, 23]. However, there is an unaddressed demand for an efficacious treatment for CKD that can impede the progression of the disease, forestall the onset of end-stage kidney disease and cardiovascular complications, and extend patient lifespans [23–25].

Diuretic medications enhance the elimination of water and electrolytes, and are prescribed across a broad spectrum of pathologies, diseases, and clinical conditions, addressing issues such as fluid overload, hypertension, and glaucoma. Their effects manifest through diverse mechanisms, frequently involving the modulation of ion transport systems along nephrons [26]. Concerns regarding the effectiveness and safety of diuretics in patients with CKD have persisted for many years. Although recent data indicate that diuretics use result in antihypertensive effects in individuals with advanced CKD [27–31], they may increase the risk of AKI or ESRD [9, 10].

In contrast to previous clinical studies, our data revealed a higher aHR of the eGFR reduction during the first 60 days, namely AKI, in patients with DM treated with SGLT2i. The findings were similar with previous reports of severe kidney events, particularly with canagliflozin and dapagliflozin use, that prompted the United States Food and Drug Administration (US FDA) to issue an AKI warning in 2016 [32]. A subsequent analysis by Perlman et al. of patients using SGLT2i through September 2016 discovered 1,224 cases of AKI events and a nearly three-fold higher incidence of AKI with the use of SGLT2i versus other medications [33]. When adjusting the risk of AKI attributable to DM, a significant (1.5-fold greater) risk remained in connection to AKI [33]. Several potential biological factors for kidney damage have been postulated, including: (1) reduced pre-renal and medullary perfusion from reduced afferent vasodilation [34, 35]; (2) volume depletion from diuresis [33, 36]; and (3) structural remodeling in proximal convoluted tubules, such as crystal formation and Armanni-Ebstein lesions, from prolonged hyperglycosuria [37, 38]. Consequently, intratubular oxidative stress is elevated and Toll-like receptors are activated, enhancing the inflammatory response.

Renal damage occurs as a result of local renal tubular injury [39]. The proposed pathomechanism was supported by analytic data from the FDA Adverse Events Reporting System, which showed that canagliflozin was responsible for around 80% of AKI cases versus empagliflozin for 13% and dapagliflozin for 7% [33]. However, because the initiation of SGLT2i is accompanied by renin-angiotensin-aldosterone system (RAAS) inhibition, which leads to intraglomerular hemodynamics and tubuloglomerular feedback, the accuracy of diagnosing AKI based on serum creatinine level increases alone has been questioned. Although adverse events (AEs) related to hypovolemia were more commonly reported in SGLT2i-treated patients, the odds of AKI events were reduced in favor of SGLT2i according to a large-scale systematic review/meta-analysis published in 2019 of 112 randomized trials and controlled observational cohort studies investigating renal AEs (including AKI, an increased serum creatinine level, a decreased eGFR, and hypovolemia-related AEs) [40]. Empagliflozin, dapagliflozin, and canagliflozin had comparable benefits on the renal AE rate [40]. In this regard, our findings contribute to the growing body of evidence demonstrating the progression of CKD in conjunction with the potential severe renal effects reported by earlier investigations.

This study has several limitations. First, not all CKD/ESRD confounding factors, such as HbA1c; baseline eGFR or CKD status; chronic heart disease and corresponding functional status; medication use, particularly RAAS inhibitors (such as angiotensin-converting enzyme inhibitors); other cardiovascular disease; and diuretic medication use, were adjusted for. Second, various age populations were included, SGLT2i usage was not stratified for the risk adjustment, and various confounders were not clarified in the results. Third, compared to other studies, the length of follow-up was rather short at < 1 year. Fourth, the diagnoses of CKD/ESRD were not stratified by disease stage. Consequently, the result may be biased, as the measured incidence of CKD/ESRD or worsening renal function may vary among individuals. Fifth, the registration of CKD/ESRD may be delayed after the initiation of SGLT2i; thus, in association with the short follow-up, the incidence and prevalence of CKD/ESRD among different conditions could be underestimated. Nonetheless, the current study still provides useful information about the correlation between SGLT2i usage and renal AEs. Finally, surveillance bias is a possibility. The level of urbanization may be linked to medical accessibility, leading to variations in the prevalence of CKD and medication administration between urban and rural areas. The Taiwanese government has implemented a single-payer, compulsory, social insurance system that covers > 99% of residents, ensuring equitable medical access between urban and rural areas through the provision of free medical care [41, 42]. As such, surveillance bias may have been mitigated. Furthermore, owing to constraints in the database, the follow-up is limited to 2017. Despite this, the available short-term data indicates a protective effect of SGLT2 inhibitors against CKD/ESRD. However, the abbreviated follow-up duration may introduce misclassification, potentially leading to an underestimation of CKD risk if development occurs after the study period. In addition, to eliminate confounding bias, the study employed the propensity score matching method for matching. Additionally, to minimize information bias and prevent subject misdiagnosis, the study defined criteria requiring at least two outpatient care visits or one hospitalization to ensure the validity of the diagnosis.

In conclusion, DM is a widely recognized risk factor for both CKD and cardiovascular disease. Thiazide diuretics are the primary choice of medication for the treatment of HT. However, the administration of diuretics can result in complications such as kidney congestion, decreased renal perfusion, reduced secretion of diuretics by renal tubules, abnormalities in the neuroendocrine system, disrupted transport of ion transporters, possible drug interactions, electrolyte imbalances, and hypoproteinemia. Results of this study revealed that diuretics can increase the risk for CKD in patients with DM; however, when combined with SGLT2i, diuretics appear to offer protection against CKD. Consequently, in clinical practice, when diuretics usage is necessary for refractory HT, heart failure, or general edema, and cannot be withdrawn even if it increases the risk for CKD, combination use of diuretics and SGLT2i may be a better strategy to decrease the risk for CKD. This research may offer valuable guidance to healthcare professionals when making medication recommendations.

## Data Availability

Data is available through the National Health Insurance Research Database (NHIRD) offered by the Taiwan National Health Insurance (NHI) Administration. Nonetheless, in compliance with legal regulations outlined in the “Personal Information Protection Act” by the Taiwan government, the data cannot be publicly disclosed. If you are interested in acquiring this data, you may initiate official requests to the NHIRD via their website (https://dep.mohw.gov.tw/DOS/lp-2506-113.html).
